# Structural Equality and Support Index in Early Childhood Education

**DOI:** 10.1001/jamanetworkopen.2024.32050

**Published:** 2024-08-30

**Authors:** Mirinda M. Morency, Arthur J. Reynolds, Marley Loveman-Brown, Rachel Kritzik, Suh-Ruu Ou

**Affiliations:** 1 Institute of Child Development, University of Minnesota, Minneapolis; 2 Human Capital Research Collaborative, University of Minnesota, Minneapolis

## Abstract

**Importance:**

Whether early childhood education is associated with a wide range of adult outcomes above and beyond individual- and family-level outcomes is unknown. As a consequence of improving educational and career success, it is postulated that participation in high quality, comprehensive programs can promote residence in more supportive community contexts in adulthood.

**Objective:**

To investigate whether participation in high-quality early childhood programs (ECP) in high-poverty neighborhoods is associated with neighborhood-level social determinants of health (SDH) at midlife.

**Design, Setting, and Participants:**

This cohort study analyzed data from the Chicago Longitudinal Study, a prospective cohort investigation following-up 989 children aged 3 to 4 years attending the Child-Parent Center (CPC) preschool program between 1983 and 1985 and a comparison group of 550 children using a nonrandomized trial design. Participants from the original sample who completed a telephone interview on health and well-being between ages 32 and 37 years were included in this analysis. Data analysis was conducted from April to June 2024.

**Exposure:**

Participation in a CPC program, which includes preschool (ages 3 to 4 years) and school-age (kindergarten through third grade), vs usual early education programs.

**Main Outcomes and Measures:**

The study used a new SDH measure (Index of Structural Equality and Support [I-SES]) based on the Healthy People 2030 framework. This 9-item index score included neighborhood-level assessment, measurement of the quality of education and health services, and assessment of racial discrimination in social and community contexts. Years of education by age 34 years was assessed as the key mediator of influence.

**Results:**

A total of 1124 individuals (mean [SD] age at survey completion, 34.9 [1.4] years; 614 women [54.6%]; 1054 non-Hispanic Black [93.8%]; 69 Hispanic [6.2%]; 1 non-Hispanic White [<0.1%]) were included in the study, of whom 740 were in the CPC cohort and 384 were in the comparison cohort. After adjustment for baseline attributes and attrition, compared with no CPC preschool, CPC preschool was associated with significantly higher mean (SD) I-SES scores (5.93 vs 5.53; mean difference, 0.40; 95% CI, 0.16-0.65; standardized mean difference = 0.22). Compared with CPC participation for 0 to 3 years, CPC participation for 4 to 6 years showed a similar pattern of positive associations (adjusted mean I-SES score, 5.97 vs 5.69; mean difference, 0.28; 95% CI, 0.06-0.50; *P* = .01; SMD = 0.15). CPC participation had a larger-magnitude association with I-SES in married vs single-parent households. Years of education partially mediated the association of CPC with I-SES (up to 41%), especially among those growing up in the highest-poverty neighborhoods.

**Conclusions and Relevance:**

This cohort study found that early childhood programming is associated with SDH in adulthood. These findings reinforce the importance of early childhood education in addressing health disparities and contributing to healthier, more equitable communities and suggest that educational attainment is a key mechanism for health promotion.

## Key Points

**Question:**

Does a comprehensive early childhood education program promote engagement in more supportive and resource-rich communities in adulthood?

**Findings:**

This cohort study that followed-up 1124 individuals from preschool age to adulthood found that participation in the Child-Parent Center early education program was associated with higher scores on the Index of Structural Equality and Support at midlife.

**Meaning:**

These findings suggest that early childhood programs can strengthen sociostructural and community supports well into adulthood.

## Teaser

This cohort study analyzes associations of early childhood education with social determinants of health among individuals who attended an early childhood program and were followed up through midlife.

## Introduction

Among early life experiences, participation in high-quality early childhood programs (ECP) is associated with a wide range of adult outcomes that include greater economic well-being, better cardiovascular and mental health, and reduced involvement in the criminal justice system.^1,2^ Due to the breadth of outcomes affected and the major role of educational success in creating cumulative advantages over time,^1,3^ ECPs that engage families intensively at multiple ecological levels may have carryover benefits to community-level social determinants of health (SDH).^4^ Whether it is neighborhood poverty or discrimination, these environmental stressors and other sociostructural factors have pervasive influences on health and well-being across the life course.^4^ Living in economically disadvantaged areas can limit access to essential resources such as quality health care and safe housing. Finally, despite the detrimental role of systemic racism and discrimination in areas such as health care, housing, and employment, studies show that Black communities greatly value education and view it as an avenue to social mobility, reflecting the importance of drawing value and satisfaction from one’s education as an SDH.^5^

One of the 5 overarching goals for Healthy People 2030^4^ is to create social, physical, and economic environments that promote attaining the full potential for health and well-being for all. Emerging research continues to explore the fundamental contributors underlying SDH as well as the health-related sequelae of these conditions, typically delineating the underlying modifiable determinants of health and grouping them according to categories like health behaviors, economic stability, physical environment, community safety, and clinical care.^6,7^ It is critical to adopt a holistic, upstream approach in SDH research to address risk and protective factors and behaviors, rather than disease outcomes, enabling the development of prevention and interventions to mitigate compounding health issues. Early childhood education is intertwined with SDH through its influence on educational attainment, nutrition, parental employment, and access to support services.^1^ Investing in high-quality programs can have far-reaching outcomes for individuals’ health and well-being, playing a vital role in addressing health disparities and promoting overall population health, which indicates the importance of investigating the association of ECPs with composite measures of SDH.

In this study, we assess, to our knowledge, for the first time whether an evidence-based, comprehensive ECP in high-poverty neighborhoods is associated with a new SDH index at midlife based on the 5-component framework of Healthy People 2030. The SDH variables proposed for this index have strong empirical bases that associate educational attainment with social mobility, which motivated us to also assess whether educational attainment mediates this association.^2,3,4,5^

## Methods

This cohort study was approved by the University of Minnesota institutional review board. Participants provided written and oral informed consent upon survey initiation. The reporting of the study follows the Strengthening the Reporting of Observational Studies in Epidemiology (STROBE) reporting guideline.^8^

### Design, Sample, and Setting

Data were analyzed from the Chicago Longitudinal Study (CLS), a prospective cohort investigation following 989 children aged 3 to 4 years attending the Child-Parent Center (CPC) preschool program between 1983 and 1985 and a comparison group of 550 children using a nonrandomized trial design.^9,10^ The comparison group participants attended the usual early education programs primarily in randomly selected schools matched to CPC locations based on poverty and neighborhood characteristics. A subset of participants from the original cohort completed a telephone interview on health and well-being between ages 32 and 37 years, which constituted our study sample. Some participants mailed in surveys. Questions concerned education, employment, health behavior, community resources, safety, and experiences of discrimination. Previous data from participants has been collected at ages 10 years, 15 to 18 years, 18 to 24 years, and 26 to 28 years.^10,11^ Participant sociodemographic data was collected from various sources, such as children, parents, teachers, and school administrative records. Race and ethnicity of participants were ascertained through self-report. Race and ethnicity categories included non-Hispanic Black, Hispanic, and non-Hispanic White. Race and ethnicity were included because it is an important attribute of the study sample for description, life circumstances, and influences.

The CLS design (eAppendix 1 in Supplement 1) is methodologically strong in that the CPC group included all children enrolled in these centers (none were excluded) and that they resided in the highest poverty-level neighborhoods.^10,11^ Any comparison group would by necessity be more advantaged in sociostructural attributes. These attributes minimize any potential selection bias. Moreover, all comparison group participants enrolled in alternative, usual treatment services (other preschool or kindergarten programs). Continuing school-age program enrollment was a combination of family and administrative selection and choice because children who moved from CPC schools left the program by definition, but schools also varied by number of years of school-age services offered (2 years vs 3 years). Assessment of key covariates and baseline attributes showed equivalence on nearly all factors, and this finding has been confirmed in many previous reports.^10,11,12,13^ This included achievement growth prior to enrollment in school-age services for program and comparison groups, suggesting equal performance between groups and no positive selection that would confound estimates of outcomes for program duration.^13,14^

### CPC Program

CPC provides comprehensive education and family support services aimed at mitigating the impacts of poverty (eAppendix 2 in Supplement 1).^3,9^ Enrichment experiences emphasize engagement at family, school, and community levels. After a half-day preschool program (3 hours, 5 days per week) at ages 3 and/or 4 years in small classes with child-teacher ratios of 17:2, CPC components in kindergarten through third grade include reduced class sizes (maximum of 25 students), teacher aides for each class, health services, continued parent involvement opportunities, and enriched classroom environments for enhancing holistic well-being, including physical health. Following 1 to 2 years of half-day preschool, services extend to third grade for a total of up to 6 years. The overarching goal is to promote school success, ultimately leading to better health and well-being over the life course. Many prior studies have documented program structures, impacts, and validity of program estimates. Findings have corroborated this goal with the hypothesis that benefits carryover to SDH.^3,5,11^

### Midlife Outcome Measure: Index of Structural Equality and Support

The Healthy People 2030 SDH framework is comprised of the following components: economic stability, neighborhood and built environment, health care access and quality, education access and quality, and social and community context.^4^ It is unique among sociostructural indexes in its focus on neighborhood-level assessment, measurement of the quality of services and experiences in community settings, and assessment of racial discrimination as part of social and community context. We created an overall measure using 9 dichotomous indicators for the 5 components called the Index of Structural Equality and Support (I-SES). The survey items make the index and were completed by both CPC and comparison participants. See eAppendix 3 and eTable 1 in Supplement 1 for detailed information on variable definitions. As a positive measure of supports at midlife, scores range from 0 to 9, with higher scores meaning greater endorsement of positive environment structures at midlife.

### Covariates

The family risk index comprised of 8 sociodemographic indicators measured by age 3 years (eg, high school dropout or income near the federal poverty level) and its squared term were also included to assess cumulative risk. Receipt of* c*hild welfare services and adverse child experiences from birth to age 5 years, whether the mother attended college, neighborhood poverty status by age 3 years, single-parent family status by age 3 years (from birth records), and self-reported chronic health conditions as assessed in the age 35-year survey were also included. Models with CPC preschool included school-age participation to adjust for the influence of later intervention.

### Statistical Analysis

Linear regressions were analyzed with inverse probability weighting (IPW) to adjust for attrition bias and 12 covariates, including baseline family socioeconomic status and neighborhood poverty (eAppendix 4 and eAppendix 5 in Supplement 1). SPSS software version 29 (IBM) was used to calculate 95% CIs, with a 2-tailed *P* < .05 set as the level of significance. Standardized mean differences (SMDs) of 0.20 denote practical significance. They are equivalent to a 15% to 20% change near the midpoint of the outcome distribution. CPC preschool and CPC preschool plus school-age participation were analyzed separately along with 3 subgroups: household structure (married vs single-parent status), multiple family risk status, and neighborhood poverty at preschool entry. The mediator was years of education completed by age 34 years . It was taken from administrative records (eg, National Student Clearinghouse) and supplemented with survey reports over time (eAppendix 6 in Supplement 1).

We also examined the distribution of scores in three categories: low (0-3), middle (4-6), and top (7-9). This reveals if group differences were similar across the full range of structural supports. SMDs were calculated at each of these levels for the model adjusted for baseline covariates and attrition.

Mediation was assessed by the difference-in-difference method (or percentage reduction). This is the mean difference in program estimates between groups without the mediator and estimates between groups with the mediator included in the model, and then divided by the unmediated program coefficient. This proportion is multiplied by 100 to denote the percentage reduction in the program group difference associated with the mediator, which is years of education completed. This approach to mediation provides conservative estimates by definition because complex indirect effects through paths of intervening mediators are not considered. However, our estimates are readily interpretable as direct contributors to understanding long-term associations. Data analysis was completed from April to June 2024.

## Results

A total of 1124 individuals (mean [SD] age at survey completion, 34.9 [1.4] years; 614 women [54.6%]; 1054 non-Hispanic Black [93.8%]; 69 Hispanic [6.2%]; 1 White [<.01%]) were included in the study, of whom 740 were in the CPC cohort and 384 were in the comparison cohort (Table 1). Of all participants, 560 (49.8%) resided in low-income neighborhoods. Participants had completed a mean (SD [range]) 12.90 (2.13 [7-22]) years of education by age 34 years, with 161 (14.3%) having received a bachelor’s degree or higher. The mean (SD) I-SES score for the entire cohort was 5.77 (1.84), with 281 cases (25.0%) with a score less than or equal to 4 and 414 cases (36.8%) with a score of 7 or greater. The unadjusted mean (SD) I-SES scores for the CPC preschool and comparison groups were 5.91 (1.84) and 5.53 (1.82), respectively, with values for continuing program group following a similar pattern. Study participants growing up in high poverty neighborhoods (>40% of residents below federal poverty level) had lower mean (SD) I-SES scores than the lower poverty group (5.73 [1.87] vs 5.82 [1.80]). At midlife, however, the differential was accentuated (mean [SD] score, 5.01 [1.87] vs 6.03 [1.76]). Table 2 shows that I-SES indicators were positively associated with educational attainment (years of education), the preeminent individual-level SDH in Healthy People 2030. The total index score had a correlation of with educational attainment (*r* = 0.21). Correlations with overall life satisfaction (*r* = 0.41) and self-rated health (*r* = 0.17) followed a similar pattern. See eTable 2 in Supplement 1 for group equivalence at age 35 years for the original CLC cohort.

**Table 1.** Group Equivalence at Follow-Up at Age 35 Years and for Original Chicago Longitudinal Study Cohort^a^

Child and family characteristics	Total participants, No. (%) (N = 1124)^b^	Program group (n = 740)	Comparison group (n = 384)	*P* value^c^	Original sample *P* value
Birthweight, mean (SD), lb	NA	6.82 (1.25)	6.74 (1.25)	.28	.26
Reside in neighborhood with ≥40% of the population at poverty line by age 5 y (child)	560 (49.8)	415 (56.0)	145 (38.0)	<.001	<.001
Family risk index score (0-8) by age 3y, mean (SD)	NA	4.42 (1.67)	4.46 (1.72)	.69	.80
Family risk index score squared, mean (SD)	NA	22.31 (14.11)	22.86 (14.48)	.54	.54
Gender					
Women	614 (54.6)	419 (56.6)	195 (50.8)	.06	.09
Men	510 (45.4)	321 (43.4)	189 (49.2)		
Race and ethnicity					
Non-Hispanic Black	1054 (93.8)	690 (93.2)	364 (94.8)	.31	.68
Hispanic	69 (6.2)	49 (6.8)	20 (5.2)		
Non-Hispanic White	1 (<0.1)	1 (<0.1)	0		
≥4 Family risk factors	802 (71.4)	529 (71.5)	273 (71.1)	.89	.64
Single-parent family status	845 (75.2)	552 (74.6)	293 (76.3)	.53	.74
Mother not employed full or part-time	730 (64.9)	486 (65.7)	244 (63.5)	.48	.50
Mother attended college	143 (12.7)	101 (14.0)	42 (11.0)	.20	.05
Any child welfare case histories	40 (3.6)	23 (3.1)	17 (4.4)	.26	.09
Chronic health condition by age 10 y	162 (14.4)	109 (14.7)	53 (13.8)	.68	.44
Original cohort with main outcome	1124 (100.0)	740 (74.8)	384 (69.8)	.04	NA
Years of education by age 34 y, mean (SD)^d^	NA	13.00 (2.14)	12.39 (2.05)	<.001	<.001

Abbreviation: NA, not applicable.

^a^ Except for chronic health conditions (retrospectively reported on the midlife survey), the baseline indicators were measured up to age 3 years or closely to time of program enrollment. The 8 family risk factors included sociodemographic indicators associated with lower child well-being (high school dropout, not employed, family income near the poverty line, single-parent status, mother younger than 20 years at child’s birth, receipt of public aid, residence in a low-income school neighborhood, and 4 or more children in the family).

^b^ The sample sizes reported in column 2 are for the endorsed (1) code for dichotomous variables.

^c^ This* P *value refers to the group difference between the program and comparison groups at the beginning of the study (baseline) and the original sample sizes of 989 and 550 participants, respectively.

^d^ As the hypothesized mediator, educational attainment is shown for description only, and was measured primarily from administrative records supplemented by survey reports.

**Table 2.** Indicators for the Index of Structural Equality and Support in the Chicago Longitudinal Study at Midlife^a^

Healthy People 2030 component and prevalence indicator^b^	Sample, No. (%) (N = 1124)	National prevalence estimate, %	Sample corrected years of education, *r*^c^
Economic stability			
1. Reside in low-poverty neighborhood (<40% below poverty level)	663 (59.0)	96.0	0.12
2. Low financial stress	667 (59.3)	40.0	0.12
Health care access and quality			
3. Have a regular doctor or place to go	888 (79.0)	83.0	0.13
4. No report of discrimination in health care	955 (85.0)	79.0	0.02
Education access and quality			
5. High satisfaction with kindergarten to 12th grade education	809 (72.0)	42.0	0.07
6. High value of education for adult life	573 (51.0)	NA	0.04
Neighborhood and built environment:			
7. High level of community safety	531 (47.2)	79.0	0.24
Social and community context			
8. High level of community support	692 (61.6)	59.0	0.09
9. Minimal report of discrimination	540 (48.0)	73.0	0.06
≥7 Indicators	414 (36.8)	NA	0.21

Abbreviation: NA, not applicable.

^a^ Mean (SD) age at midlife was 34.9 (1.4) years.

^b^ Prevalence values were rounded to match national estimates. See eTable 1 in Supplement 1 for complete descriptions of indictors and sources for national prevalence rates, which are estimated to match the Chicago Longitudinal Sample information as closely as possible for the years 2014 to 2016. Except for low-poverty neighborhood (census data), indicators are from the midlife interview and survey.

^c^ Years of education completed by age 34 years (administrative records supplemented with self-reports in adulthood).

Table 3 shows that after adjusting for baseline characteristics including early family and social environments, compared with no CPC, CPC preschool was associated with a significantly higher mean I-SES score (5.93 vs 5.53; mean difference, 0.40; 95% CI, 0.16-0.65; *P* = .03; SMD = 0.22). A similar pattern of differences was found for adjusted mean I-SES scores for CPC preschool plus school-age participation (4 to 6 years) compared with 0 to 3 years of participation (5.97 vs 5.69; mean difference, 0.28; 95% CI, 0.06-0.50; *P* = .01; SMD = 0.15).

**Table 3.** I-SES Mean Outcomes at Age 35 Years for CPC Program and Comparison Groups in the Chicago Longitudinal Study Adjusted for Early Childhood Baseline Attributes and Attrition via Inverse Propensity Score Weighting^a^

Outcome (sample group)	CPC PS I-SES score, adjusted mean (SD)^b^	Comparison group ISES score, adjusted mean (SD)^c^	Mean difference (95% CI)	Standardized mean difference^d^	CPC PS + SA ISES-score, adjusted mean (SD)^e^	Comparison group I-SES score, adjustedmean (SD)^f^	Mean difference (95% CI)	Standardized mean difference^d^	Group difference explained by education (PS vs PS+SA)^b^^,^^e^
Total score (n = 1124)	5.93	5.53	0.40 (0.16 to 0.65)^g^	0.22	5.97	5.69	0.28 (0.06 to 0.50)^g^	0.15	16% vs 18%
Household structure									
Single-parent (n = 845)	5.65	5.56	0.09 (−0.06 to 0.62)	0.05	5.84	5.65	0.19 (−0.07 to 0.44)	0.10	25% vs 29%
Married (n = 279)	6.36	5.42	0.94 (0.46 to 1.44)^h^	0.51	6.31	5.80	0.51 (0.08 to 0.93)^g^	0.28	9% vs 7%
Neighborhood poverty^i^									
Higher (≥40%) (n = 560)	5.75	5.45	0.30 (−0.06 to 0.67)	0.16	5.86	5.64	0.22 (−0.10 to 0.54)	0.12	31% vs 41%
Lower (<40%) (n = 564)	6.07	5.58	0.49 (0.14 to 0.84)^g^	0.27	6.07	5.73	0.34 (0.03 to 0.65)^g^	0.18	11% vs 8%
Family risk status (8 demographic indicators)									
Higher risk (n = 802)^j^	5.91	5.47	0.44 (0.13 to 0.71)^g^	0.24	5.90	5.65	0.25 (−0.06 to 0.76)	0.14	19% vs 27%
Lower risk (n = 322)	5.98	5.68	0.30 (−0.20 to 0.77)	0.16	6.17	5.80	0.37 (−0.03 to 0.50)	0.20	18% vs 9%

Abbreviations: CPC, child-parent center; I-SES, Index of Structural Equality and Support; PS, preschool; SA, school age.

^a^ I-SES scores range from 0 to 9. The share of the group difference explained by educational attainment (years of education by age 34) is calculated as the change in the group difference from the unmediated to mediated model divided by the unmediated group difference multiplied by 100.

^b^ PS was defined as any CPC PS for 1 or 2 years (ages 3 and 4 years; part-day).

^c^ Comparison group was those with no PS.

^d^ Standardized difference is based on the within-group SD of the total sample for each program contrast; both had an SD of 1.84).

^e^ PS+SA was the full CPC program, which included 1 or 2 years of preschool and continuing up to second or third grade for 4 to 6 years of services.

^f^ The comparison group is all those with fewer years (0 to 3) and includes the non-CPC comparison group.

^g^ 95% CI does not include 0 (2-tailed *P* < .05).

^h^ 95% CI does not include 0 for interaction test between subgroups (2-tailed *P* < .05).

^i^ Neighborhood poverty was measured from the 1980 census track databased on home address records by age 3 years (1982-1983). It is the percentage of residents at or below the federal poverty level.

^j^ High family risk is defined as the presence of 4 or more risk factors (eg, single-parent status or school dropout) vs 0 to 3 factors assessed by age 3 years.

The eFigure in Supplement 1 shows the pattern of adjusted program group differences (SMDs) at low, middle, and top categories of the I-SES distribution. For the total sample, X participants (12.4%) were in the low category, X (50.8%) in the middle category, and X (37.X%) in the top category. For the CPC preschool vs none contrast, program participants were more likely to be in the top group of I-SES scores of 7 to 9 of 9 points (SMD = 0.25). They were less likely to be in the lower 2 groups (CPC preschool, SMD  = −0.12; no CPC, SMD = −0.23). The pattern was similar for the dosage groups (4-6 years vs 0-3 years). When separated by neighborhood poverty status at the time of program participation, children in CPC growing up in relatively lower poverty settings (<40% of residents below poverty) experienced the largest benefits in I-SES. For the top score group, SMDs were 0.33 and 0.25, respectively, for CPC preschool vs none and higher vs lower dosage groups (eFigure in Supplement 1).

Subgroup findings overall showed similar associations across groups, but there were larger-magnitude associations among more advantaged groups. One significant subgroup interaction was identified. CPC preschool was had a larger-magnitude association with I-SES in married households (adjusted mean score, 6.36 vs 5.42; mean difference, 0.94; 95% CI, 0.46 to 1.44; *P* < .001; SMD = 0.51) than in single-parent households (adjusted mean score, 5.65 vs 5.56; mean difference, 0.09; 95% CI, −0.06 to 0.62; *P* = .67; SMD = 0.05). This pattern was also found for the dosage groups of 4 to 6 years vs fewer years (Table 3). Similarly, the lower neighborhood poverty group had significantly higher adjusted mean I-SES scores, including both the preschool vs comparison contrast (6.07 vs 5.58; mean difference, 0.49; 95% CI, 0.14 to 0.84; *P* = .02; SMD = 0.27) and preschool plus school age vs comparison contrast (6.07 vs 5.73; mean difference, 0.34; 95% CI, 0.03 to 0.65; *P* = 0.1; SMD = 0.18). The lone comparison favoring higher risk groups was for family risk status, which was found in both the preschool vs comparison contrast (adjusted mean I-SES score, 5.91 vs 5.47; mean difference, 0.44; 95% CI, 0.13 to 0.71; SMD = 0.24) and preschool plus school age vs comparison contrast (adjusted mean I-SES score, 5.90 vs 5.65; mean difference, 0.25; 95% CI, −0.06 to 0.76; *P* = X.X; SMD = 0.14). No differences for the full program groups were detected (Table 3).

Alternative estimates support robustness (eAppendix 7 and eTable 3 in Supplement 1). Estimated program outcomes were similar between IPW and non-IPW models, suggesting that attrition occurred at random and was not associated with baseline characteristics (eAppendix 6 in Supplement 1).

For the mediation results for the total sample, years of education accounted for 16% to 18% of the association of CPC with I-SES. Mediation increased with economic disadvantage. Among those growing up in the highest poverty neighborhoods, 31% to 41% of the association was mediated by years of education (Table 3). These values are above and beyond the influence of baseline characteristics and program participation. These results reflect only the direct association with educational attainment. More complex associations are possible. Robustness testing using different model specifications did not alter the pattern of findings and was consistent with results reported here (eTable 3 in Supplement 1).

## Discussion

The findings of this cohort study provide evidence suggesting that a multilevel, comprehensive-service ECP is associated with SDH in adulthood. To our knowledge, this is the first study to find such an association. Findings also document that this new index measure of SDH comprised of impactful neighborhood indicators can discriminate between the early life experiences of children who do or do not participate in intensive educational enrichment. The findings also establish that the benefits of ECPs extend beyond individual-level education and occupational success to the broader sociostructural environment. Although in general CPC participation had similar positive associations with I-SES across subgroups, the SMD for children growing up in married households exceeded those in single-parent households and in other subgroups by a factor of 2 or higher. This finding suggests that economic and family resources available in the early years of life create cumulative advantages that are unlikely to be overcome by social intervention alone, even comprehensive programs like CPC. Concerted efforts at multiple levels over extended periods of time, however, can improve well-being.

The finding that educational attainment, a leading individual-level SDH, accounted for a sizable share of observed differences for the most economically disadvantaged groups suggests that educational success is one mechanism for reducing disparities in supportive social environments. This finding is consistent with a large body of research demonstrating that ECPs have compensatory and protective effects for children and families growing up in the most economically disadvantaged communities.^1,2,9^ However, only programs high in quality have these benefits, and the barriers to such quality have increased in recent years.

Nevertheless, the developmental origins of educational attainment are complex and involve socioeconomic position, home and school environments, motivational and socioemotional influences, and achievement behaviors.^3,11,15^ Investigation of these and related influences were beyond the scope of the present study. Previous findings in the CLS and related studies show that the cumulative advantages initiated by ECPs are complex and circuitous, including individual and personal, family, school, and community processes.^3,11^ The early cognitive and scholastic advantages of CPC, for example, carryover to strengthened parental involvement in school, enrollment in higher quality schools, avoidance of delinquent behaviors, and ultimately higher educational attainment.^3,11^ This process and others warrant further investigation and confirmation, especially in comprehensive frameworks such as the 5-Hypothesis Model.^3^

### Limitations

This study has limitations. The main limitation is that our SDH measure, although broad and based on a well-documented framework, may not fully represent community and structural influences. Moreover, results are correlational and warrant replication.

## Conclusions

This cohort study found that ECP was associated with SDH in adulthood. These findings suggest that CPC and similar programs can contribute to broader efforts to mitigate health disparities and create healthier, more equitable communities. Educational attainment appears to be a key transmitter of observed benefits, which reinforces its importance as a major goal of ECPs.

## Notes

Supplement 1. **eAppendix 1.** CLS Background **eAppendix 2**. CPC Program Description **eAppendix 3.** SDH Definitions and Justification **eTable 1.** Index of Structural Equality and Support (I-SES) as Operationalized in Chicago Longitudinal Study **eAppendix 4.** Covariates in Model Specification **eAppendix 5.** Inverse Probability Weighting **eAppendix 6.** Educational Attainment Mediator **eTable 2.** Group Equivalence at Age 35 Follow Up and for Original Chicago Longitudinal Study Cohort (N=1,124) **eFigure. **Standardized Mean Differences for 2 Child-Parent Center (CPC) Program Contrasts for Low (0-3), Middle (4-6), and Top (7-9) Scores on the I-SES [Index of Structural Equality and Support] for the Total Sample and by Neighborhood Poverty Status (40% or More vs. Less in Poverty by Child’s Age 3 years) as Assessed at Midlife **eAppendix 7.** Alternative Model Estimates **eTable 3.** Alternative Models for CPC Preschool Participation and Index of Structural Equality and Support (I-SES) at Midlife
eReferences.

Supplement 2.
Data Sharing Statement
