# Supplementary material for: Structural Equality and Support Index in Early Childhood Education
Source: JAMA Netw Open. 2024 Aug 30;7(8):e2432050. doi: 10.1001/jamanetworkopen.2024.32050 (PMC11364995; doi:10.1001/jamanetworkopen.2024.32050)
Supplement: Supplement 1. — eAppendix 1. CLS Background eAppendix 2. CPC Program Description eAppendix 3. SDH Definitions and Justification eTable 1. Index of Structural Equality and Support (I-SES) as Operationalized in Chicago Longitudinal Study eAppendix 4. Covariates in Model Specification eAppendix 5. Inverse Probability Weighting eAppendix 6. Educational Attainment Mediator eTable 2. Group Equivalence at Age 35 Follow Up and for Original Chicago Longitudinal Study Cohort (N=1,124) eFigure. Standardized Mean Differences for 2 Child-Parent Center (CPC) Program Contrasts for Low (0-3), Middle (4-6), and Top (7-9) Scores on the I-SES [Index of Structural Equality and Support] for the Total Sample and by Neighborhood Poverty Status (40% or More vs. Less in Poverty by Child’s Age 3 years) as Assessed at Midlife eAppendix 7. Alternative Model Estimates eTable 3. Alternative Models for CPC Preschool Participation and Index of Structural Equality and Support (I-SES) at Midlife eReferences. [file jamanetwopen-e2432050-s001.pdf]

Supplemental Online Content

Morency MM, Reynolds AJ, Loveman-Brown M, Kritzik R, Ou S-R. Structural equality and support index in early childhood education. JAMA Netw. Open. 2024;7(8):e2432050. doi:10.1001/jamanetworkopen.2024.32050

eAppendix 1. CLS Background
eAppendix 2. CPC Program Description
eAppendix 3. SDH Definitions and Justification
eTable 1. Index of Structural Equality and Support (I-SES) as operationalized in Chicago Longitudinal Study
eAppendix 4. Covariates in Model Specification
eAppendix 5. Inverse Probability Weighting
eAppendix 6. Educational Attainment Mediator
eTable 2. Group Equivalence at Age 35 Follow Up and for Original Chicago Longitudinal Study Cohort (N=1,124)
eFigure. Standardized Mean Differences for 2 Child-Parent Center (CPC) Program Contrasts for Low (0-3), Middle (4-6), and Top (7-9) Scores on the I-SES [Index of Structural Equality and Support] for the Total Sample and by Neighborhood Poverty Status (40% or More vs. Less in Poverty by Child's Age 3 years) as Assessed at Midlife
eAppendix 7. Alternative Model Estimates
eTable 3. Alternative Models for CPC Preschool Participation and Index of Structural Equality and Support (I-SES) at Midlife
eReferences

This supplemental material has been provided by the authors to give readers additional information about their work.

© 2024 Morency MM et al. *JAMA Network Open.*

eAppendix 1. CLS Background

The Chicago Longitudinal Study (CLS) is an investigation of the effects of Child-Parent Center Education Program (CPC).[1] The CLS follows a same-age cohort of low-income children (N = 1539, 93% Black/African American, 7% Hispanic/Latino) born in 1979-1980 who participated in government-funded early education programs beginning in preschool and kindergarten (1983-1986) in the Chicago Public School District. Participants grew up in high-poverty neighborhoods in central-city Chicago and either participated in one of 20 CPC preschool and kindergarten programs (N = 989) or a matched comparison of either randomly selected CPS kindergarten programs or one of 8 CPC programs without a preschool component (N = 550). CPC participants continued in the program through 2nd or 3rd grade.

The primary aims of the study are to: (1) evaluate the impact of the CPC program on child and family development, (2) identify and better understand the pathways through which the effects of program participation influence long-term health and well-being, and (3) investigate contributions of individual, family, school, and community environments from early childhood to midlife.[2]

As a prospective cohort design, participants have been followed throughout life and are currently between 43 and 44 years of age. A wide range of predictors and outcomes that may be related to high-quality early childhood education, including parent involvement, social-emotional learning, educational attainment, employment and income, and criminal justice involvement, among others have been assessed using various modes of data collection, including birth records, K-12 school records, parent and teacher report, participant report, and in-depth health exams for select participants. Previous data has been collected at ages 10, 15 to 18, 18 to 24, and 26 to 28.

The current phase of the CLS aims to understand the association between CPC participation and physical health and well-being outcomes in midlife. Nearly 1124 participants (73%) from the original sample completed a written and telephonic survey on health and well-being between ages 35 and 37. This study uses data obtained from the midlife survey to devise an index for measuring social determinants of health based on participants' self-report of economic stability, health care access/quality, education access/quality, neighborhood and built environment, and social and community context.

© 2024 Morency MM et al. *JAMA Network Open.*

eAppendix 2. CPC Program Description

The Child-Parent Center (CPC) Program was established in 1967 through funding from Title I of the landmark Elementary and Secondary Education Act of 1965. Initially implemented in four sites in Chicago and later expanded to 25, the program originally served families in high-poverty neighborhoods that were not reached by other early childhood programs.[3] In 2012, xxxxxx at the University of Minnesota, along with participating districts, revised the CPC Program as a comprehensive school reform model to serve children in a broad variety of geographic and economic contexts. Under an Investing in Innovation Grant from the U.S. Department of Education, Human Capital Research Collaborative (HCRC) began an expansion of the CPC program in 2012 in four school districts, including St. Paul, Minnesota, and Chicago, Evanston, and Normal in Illinois.[4]

CPC is designed to enhance school readiness and achievement, promote parent involvement and engagement in the school and community, and enhance socio-emotional learning with an emphasis on self-control, self-efficacy, and personal responsibility. Breakfasts and lunches are provided, school nurses work with families on site, and referrals to health centers, speech therapy and other supports are provided. CPCs are in a stand-alone school or center in which all children receive services. After 1 or 2 years of part day preschool, kindergarten through third grade services are provided.

Key elements of the CPC program design include collaborative leadership, effective learning experiences, aligned curriculum, parent involvement, professional development, and continuity. Continual research efforts on the CPC P-3 program and its six core elements have demonstrated effectiveness and scalability of the intervention. Impacts to date demonstrate that CPC participation has the potential to influence various social determinants of health, improving the conditions and environments that are linked to positive health outcomes over the life course. When implemented with fidelity, these core elements are expected to initiate a trajectory of upward mobility among disadvantaged populations. Prior findings suggest influence through critical pathways which include increased family support behavior, better school quality, cognitive advantage and educational attainment, and reduced criminal justice involvement.

© 2024 Morency MM et al. *JAMA Network Open.*

eAppendix 3. SDH Definitions and Justification

Table 1 provides the operational definitions of the nine indicators of the social determinants of health (SDH) that make up the Index of Structural Equality and Support (I-SES). Correlations of each indicator with educational attainment are also provided. Broadly defined, SDH are the non-medical factors that influence health. Healthy People 2030 considers SDH to be "the conditions in the environments where people are born, live, learn, work, play, worship, and age" as well as the network of forces beyond medical care that influence these conditions.[5] According to the Healthy People 2030 framework, SDH can be grouped into 5 domains: education access/quality, economic stability, neighborhood/built environment, social/community context, and health care access/quality. The study of SDH serves to illustrate that health is not solely determined by genetics or individual behavior but is heavily influenced by lived experience. In recent years, public health research has sought to elucidate the mechanisms through which SDH drive health disparities as well as potential policies and practices that could address longstanding inequities. It is critical to adopt a wholistic, upstream approach in SDH research to address risk and protective factors and behaviors, rather than disease outcomes, enabling the development of prevention and interventions to mitigate compounding health issues. Early childhood education is intertwined with social determinants of health through its influence on educational attainment, nutrition, parental employment, and access to support services.[6-9] Investing in high-quality ECE can have far-reaching effects on individuals' health and well-being, playing a vital role in addressing health disparities and promoting overall population health, which indicates the importance of investigating the link between early childhood education interventions and composite measures of social determinants of health.

Many indices, databases, and tools have been developed to measure and describe SDH, often comprised of key domains and subdomains with significant variability in number and type of indicators measured. Some of the existing indices are narrowly defined to address a specific research question and their comprising measures are carefully selected to capture factors uniquely tied to the outcome of interest. Measures of economic status vary greatly across indices with each including a different combination of indicators of employment, income, and opportunity.[10]

eTable 1. Index of Structural Equality and Support (I-SES) as Operationalized in Chicago Longitudinal Study (CLS) at Age 35

**Metric**	**CLS Measure, Mean Age 35 y/o**	**Evidence of Validity: Correlation with Highest Grade Completed**	**National Prevalence Estimate**
A. Economic stability			
Financial stability (low financial stress)	6 items: Financial stability scale (recoded to dichotomous items, 0-6 score range; mean = 3.77, SD = 1.52). Low financial stress = 4 to 6 score points).<br>1) I find it difficult to live on my total household income right now. (1-Agree strongly, 6-Disagree strongly)<br>2) I am confident today that my family and I will be adequately housed, fed, and cared for medically over the next 6 months. (1-Agree strongly, 6-Disagree strongly)<br>3) I expect in the next 2 months that I will have to reduce my standard of living. (1- Agree strongly, 6-Disagree strongly)<br>4) How much financial strain on your budget is paying your rent or mortgage each month? (1-No strain at all, 5-Significant strain)<br>5) How worried are you that you will have difficulty making your rent or mortgage payments over next year? (1- Not at all worried, 4-Very worried)<br>6) Have you experienced family financial problems over the course of your life? (Yes/No) If yes, how old were you when this happened?	r = .122, p < .001	Rate (2018): 40%<br><br>Hasler, A., Lusardi, A., & Valdes, O. (2021). Financial anxiety and stress among US households: New evidence from the national financial capability study and focus groups. *FINRA Investor Education Foundation*.
Reside in low poverty neighborhood	United States Census Bureau. *2010 Census*	r = 124, p < .001	Rate (2010-2014): 96%<br><br>Kneebone, E., & Holmes, N. (2016, March 31). *U.S. concentrated poverty in the wake of the Great Recession*. Brookings Institute.

© 2024 Morency MM et al. *JAMA Network Open.*

			https://www.brookings.edu/articles/u-s-concentrated-poverty-in-the-wake-of-the-great-recession/
B. Health care access/quality			
Regular doctor or place to go	1 item:<br>Do you have a regular doctor or place you go to for health care for yourself?	r = .126, p < .001	Ratte (2022): 83%<br><br>National Center for Health Statistics. Percentage of having a doctor visit for any reason in the past 12 months for adults aged 18 and over, 2022. National Health Interview Survey.
No discrimination in health care	1 item:<br>Have you experienced any of the following forms of discrimination in your life because of your race, ethnicity, gender, age, religion, physical appearance, or other characteristics? Denied medical care or received inferior medical care.	r = .021, p = .491	Rate (2019): 79%<br><br>Nong, P., Raj, M., Creary, M., Kardia, S. L., & Platt, J. E. (2020). Patient-reported experiences of discrimination in the US health care system. *JAMA network open*, *3*(12), e2029650-e2029650.
C. Education access/quality			
High satisfaction with K-12 education	1 item:<br>How satisfied are you with your education during the following years? (K 12th grade, averaged)	r = .066, p = .030	Rate (2022): 42%<br><br>Saad, L. (2022, September 1). *Americans' satisfaction with K-12 education on Low Side*. Gallup. https://news.gallup.com/poll/399731/americans-satisfaction-education-low-side.aspx
High value of education for adult life	1 item:<br>How valuable was your K-12th grade education in preparing you for adult life?	r = .043, p = .159	N/A
D. Neighborhood & built environment			

© 2024 Morency MM et al. *JAMA Network Open.*

High level of community safety	3 items (4-point scale, range 3-12; mean = 7.43, SD = 2.01; high community safety = 8 to 12 score points):<br>1) How would you describe the physical condition of the dwelling where you currently live<br>2) How would you describe the safety of the neighborhood where you currently live<br>3) Overall, does your current housing situation meet your needs	r = .235, p < .001	Rate (2022): 79%<br><br>NPR, Robert Wood Johnson Foundation, & Harvard T.H. Chan School of Public Health. (2022, August 1). *Personal Experiences of U.S. Racial/ Ethnic Minorities in Today's Difficult Times*. NPR-RWJF-Harvard Poll. https://legacy.npr.org/assets/pdf/2022/08/NPR-RWJF-Harvard-Poll.pdf
E. Social & community context			
High level of community support	3 items (6-point scale from strongly agree to strongly disagree; range = 3-18; mean = 13.14, SD = 4.37; high community support = 13 to 18 score points):<br>1) I don't feel I belong to anything I'd call a community.<br>2) I feel close to other people in my community.<br>3) My community is a source of comfort.	r = .093, p = .002	Rate (2018): 59%<br><br>Parker, K., Horowitz, J., Brown, A., Fry, R., Cohen, D., & Igielnik, R. (2018, May 22). *5. Americans' satisfaction with and attachment to their communities*. Pew Research Center. https://www.pewresearch.org/social-trends/2018/05/22/americans-satisfaction-with-and-attachment-to-their-communities/
No or one report of discrimination	<1 endorsement of discriminatory experience in the lifetime	r = -.056, p = .069	Rate (2020): 73%<br><br>Kaiser Family Foundation. (2022, June). *KFF Health Tracking Poll – June 2020*. Henry J Kaiser Family Foundation. https://files.kff.org/attachment/Topline-KFF-Health-Tracking-Poll-June-2020.pdf
I-SES, 0-9	Composite score	r = .212, p < .001	N/A

Note. I-SES sample=1124. Highest grade completed=years of education by age 34.

© 2024 Morency MM et al. *JAMA Network Open.*

eAppendix 4. Covariates in Model Specification

The 12 baseline characteristics included in the model specification were as follows, and are consistent with many previous studies.[1-4] These included children's race (93% Black), gender, and birthweight from county/state records. The family risk index comprised of eight sociodemographic indicators measured by age 3 (e.g., high school dropout, income near the federal poverty level) and its squared term were also included to assess cumulative risk. Child welfare services and adverse child experiences from birth to age 5, whether mother's attended college, neighborhood poverty status by age 3, single-parent family status by age 3 (from birth records), and self-reported chronic health conditions as assessed in the age 35 survey. Apart from the dosage/duration model, participation in the CPC school-age program from first to third grade was also included to remove the influence of later program services. Table 2 shows the equivalence of the CPC program and comparisons groups at follow up and at the beginning of the study by covariate and background characteristics.

eAppendix 5. Inverse Propensity Weighting

Because almost 30% of the original cohort did not participate in the midlife survey, Inverse Propensity Score Weighting (IPW) was used to adjust for potential attrition bias. IPW creates a pseudo-population in which attrition from the study sample is independent of baseline characteristics. This removes potential confounding and improves statistical inference, making those included in the analysis comparable to the original study population. Using logistic regression, 31 baseline variables related to birth outcomes and demographics (BD), home environment (HE), program factors (PR), and school and neighborhood factors (SN), were used to estimate probabilities (propensities) of sample recovery (SR) with the following model equation using an attrition weight variable:

Wi = 1/SR
SR = Constant + Bj1BD + Bj2HE + Bj3PR + Bj4SN + e

The weights generated were applied to subsequent outcome analyses. IPW works so that participants with higher weights are counted more heavily in program effect estimates, because they have lower probabilities of responding to the midlife survey. Participants with higher probabilities of responding and therefore lower weights were counted less. However, estimated program effects were similar between IPW and non-IPW models, suggesting that attrition occurred at random and did not relate to baseline characteristics.

For the logit regression model predicting SR in the total sample, the three strongest predictors were race (Black participants were less likely to complete the midlife interview/survey), sex/gender (women were more likely than men to complete interview/survey), and socio-emotional adjustment by age 9 (third grade; participants with higher teacher ratings of socio-emotional learning were more likely to complete the interview/survey). These results were robust across different prediction models. Different prediction models also yielded nearly identical SR probabilities used to calculate the weights.

eAppendix 6. Educational Attainment Mediator

Years of education completed by age 34 was the mediator of the CPC to I-SES association. Meausured through school and administrative records, primarily the National Student Clearinghouse to which nearly all colleges and universities report, values ranged from 8 to 20administrative records. Records were supplemented with survey reports at various ages. Educational attainment is widely considered one of the most important individual level social determinant of health and predictor of well-being in adulthood.

© 2024 Morency MM et al. *JAMA Network Open.*

eTable 2. Group Equivalence at Age 35 Follow Up and for Original Chicago Longitudinal Study Cohort (N=1,124)

Child and Family Characteristics	***Prog. Group (n=740)***	***Comp. Group (n=384)***	***P-value***	***Original Sample P-value***
Birthweight in pounds	6.82	6.74	0.28	0.26
Reside in neighborhood ≥40% pop. at poverty line by child's age 5 (%)	56.0	38.0	<.001	<.001
Family risk index (0-7) by age 3	4.42	4.46	0.69	0.80
Family risk index squared	22.31	22.86	0.54	.54
Women (%)	56.62	50.78	0.06	0.09
Black (%)	93.24	94.79	0.31	0.68
Four or more family risk factors (%)	71.5	71.1	0.89	0.64
Single parent family status (%)	74.6	76.3	0.53	0.74
Mother not employed full/part-time (%)	65.7	63.5	0.48	0.50
Mother attended college (%)	14.0	11.0	0.20	.05
Any child welfare case histories (%)	3.11	4.43	0.26	0.09
Chronic health condition by age 10 (%)	14.7	13.8	0.68	0.44
Original cohort with main outcome (%)	74.8	69.8	.04	n/a
Years of education by age 34 (mediator)	13.0	12.4	<.01	<.01

Note. Except for chronic health conditions (retrospectively reported on the midlife survey), the baseline indicators were measured up to age 3 or closely to time of program enrollment. The eight family risk indicators include socio-demographic indicators (e.g., high school dropout, not employed, family income near the poverty line) associated with lower child well-being. As the hypothesized mediator, educational attainment is shown for description only.

© 2024 Morency MM et al. *JAMA Network Open.*

eFigure. Standardized Mean Differences for 2 Child-Parent Center (CPC) Program Contrasts for Low (0-3), Middle (4-6), and Top (7-9) Scores on the I-SES [Index of Structural Equality and Support] for the Total Sample and by Neighborhood Poverty Status (40% or More vs. Less in Poverty by Child's Age 3 years) as Assessed at Midlife

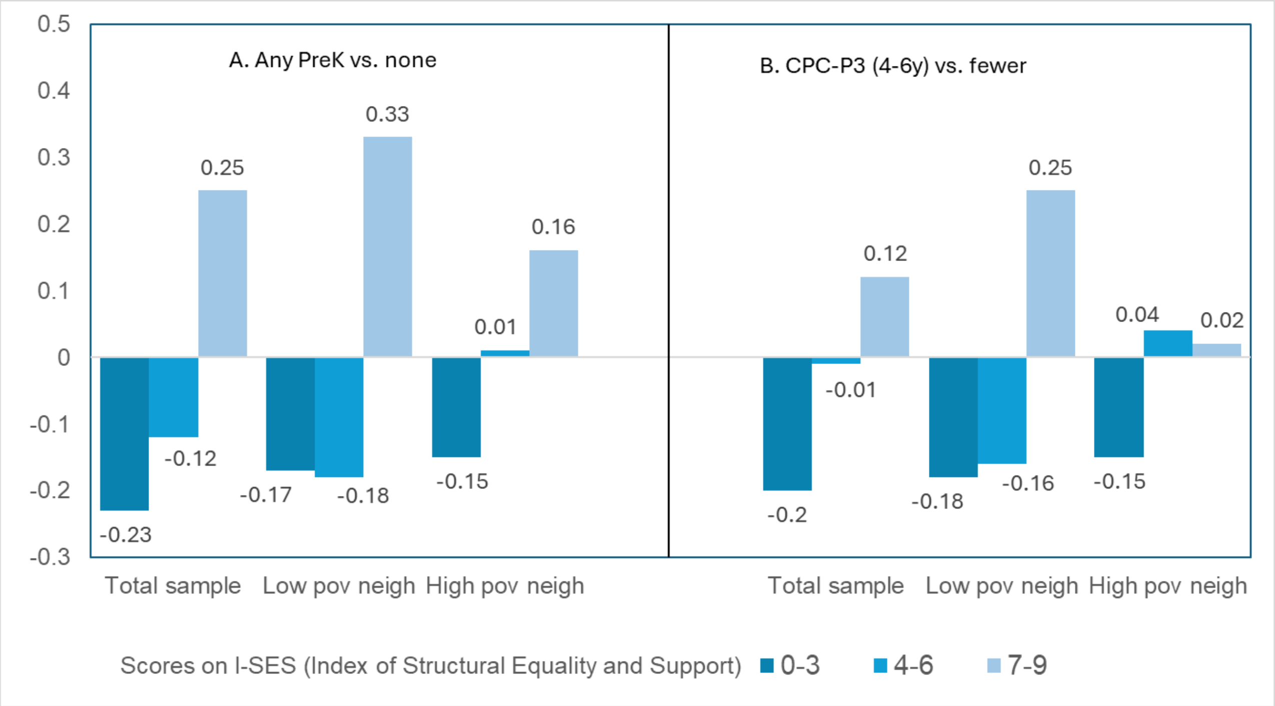

Values of 0.2 in absolute value are practically significant. 95% CI does not include 0 only for values >0.24. CPC-P3 = Child-Parent Center program from preschool to 3rd grade.

© 2024 Morency MM et al. *JAMA Network Open.*

eAppendix 7. Alternative Model Estimates

Alternative models with different assumptions and definitions of social determinants of health were explored to assess the robustness of findings from the main model (Model 2). Results in eTable 3 demonstrate that, in each instance, the adjusted group differences maintained their significant associations. To elaborate, even when incorporating socioemotional learning and achievement variables during elementary school, the significant associations of adjusted group differences were consistently sustained.

Further robustness testing was done to examine the program associations across the range of I-SES. Standardized mean differences (SMD) were calculated for three categories of scores: low (0-3), middle (4-6), and top (7-9). The same model of covariates and adjustments for potential attrition bias was used. The eFigure shows the pattern of adjusted program group differences (SMDs) at low, middle, and top categories of the I-SES distribution. For the total sample, the respective percentages for each group were 12.4%, 50.8%, and 37%. For the CPC preschool vs none contrast, program participants were more likely to be in the top group of I-SES scores of 7 to 9 of out 9 score points (SMD = .25). They were less likely to be in the lower two groups (SMDs = -0.12, -0.23). The pattern was similar for the dosage groups (CPC-P3, 4-6 years vs. 0-3 years). When separated by neighborhood poverty status at the time of program participation, CPC children growing up in relatively lower poverty settings (<40% of residents below poverty) experienced the largest benefits in I-SES. For the top score group, SMDs were 0.33 and 0.25, respectively, for CPC preschool vs. none and higher vs. lower dosage groups.

© 2024 Morency MM et al. *JAMA Network Open.*

eTable 3. Alternative Models for CPC Preschool Participation and Index of Structural Equality and Support (I-SES) at Midlife (Mean age=34.9 years; N = 1124)

**Summary statistics/model specifications**	**M0**	**M1**	**M2**	**M3**	**M4**	**M5**
**Group differences, I-SES**	**.397**	**.350**	**.396**	**.337**	**.371**	**.358**
**Standard error**	**.125**	**.126**	**.126**	**.126**	**.129**	**.128**
**P-value**	**<.01**	**.01**	**<.01**	**.01**	**<.01**	**.01**
**Standardized mean difference**	**.216**	**.190**	**.215**	**.183**	**.202**	**.195**
Unadjusted I-SES Mean (SD=1.83)	5.77	--	--	--	--	--
Model 0: No covariates/unadjusted	YES	NO	NO	NO	NO	NO
Model 1: Baseline covariates	NO	YES	YES	YES	YES	YES
Model 2: IPW attrition adjustment (Study main model)	NO	NO	YES	YES	YES	YES
Model 3: Adds in socioemotional learning	NO	NO	NO	YES	NO	YES
Model 4: Adds in school achievement	NO	NO	NO	NO	YES	YES
Model 5: Model 3 + Model 4	NO	NO	NO	NO	NO	YES

Note. The group difference is the CPC mean minus the comparison group mean, with the standardized mean differences based on the within-group standard deviation. School achievement variables include reading and math achievement test scores in kindergarten using the Iowa Tests of Basic Skills. Socioemotional learning is teacher ratings of classroom adjustment averaged over first to third grades (e.g., gets along well with others, follows directions). By design, models 3 -5 are "over-controlled" as these indicators are affected by CPC participation and their inclusion removes their contribution from the estimated program effect (association). Each model assumption is displayed. IPW=inverse propensity score weighting. I-SES=index of structural equality and support. The mean (SD) I-SES score for the original sample of 1,539 is 5.67 (1.76) with imputation.

© 2024 Morency MM et al. *JAMA Network Open.*

eReferences

1. Reynolds, A. J., & Ou, S. (2010). (Eds.). Early childhood to young adulthood: Intervention and alterable influences on well-being from the Chicago Longitudinal Study. *Children and Youth Services Review, 32*(8).

2. Reynolds, A. J., Ou, S., & Temple, J. A. (2018). A multi-component preschool to 3rd grade preventive intervention and educational attainment at 35 years of age. *JAMA Pediatrics*, 172*(3),* 247-256.

3. Reynolds, A. J., & Human Capital Research Collaborative. (2016). *Child-Parent Center Preschool to Third Grade (P-3) Program Manual*. Minneapolis: University of Minnesota, Human Capital Research Collaborative.

4. Reynolds A. J., Ou S-R., Eales L., Mondi C. F., & Giovanelli A. (2021). Assessment of a Comprehensive Early Childhood Education Program and Cardiovascular Disease Risk in Midlife. JAMA Netw Open. 2021 Aug 2;4(8):e2120752. doi: 10.1001/jamanetworkopen.2021.20752.

5. U.S. Department of Health and Human Services. (2020). Social determinants of health. Healthy People 2030. https://health.gov/healthypeople/priority-areas/social-determinants-health

6. Morrissey, T. W. (2016). Child care and parent Labor Force Participation: A review of the research literature. *Review of Economics of the Household*, *15*(1), 1–24. https://doi.org/10.1007/s11150-016-9331-3

7. Ou, S.-R., & Reynolds, A. J. (2006). Early Childhood Intervention and Educational Attainment: Age 22 Findings From the Chicago Longitudinal Study. *Journal of Education for Students Placed at Risk*, 11(2), 175–198. https://doi.org/10.1207/s15327671espr1102_4

8. Hurley, K. M., Yousafzai, A. K., & Lopez-Boo, F. (2016). Early child development and nutrition: A review of the benefits and challenges of implementing integrated interventions. *Advances in Nutrition*, *7*(2), 357–363. https://doi.org/10.3945/an.115.010363

9. Hinnant, L., Hairgrove, S., Kane, H., Williams, J., & Cance, J. D. (2022). Social Determinants of Health: A review of publicly available indices. *Research Triangle Park (NC): RTI Press*. https://doi.org/10.3768/rtipress.2022.op.0081.2212

10. Hood, C. M., Gennuso, K. P., Swain, G. R., & Catlin, B. B. (2016). County Health Rankings: Relationships Between Determinant Factors and Health Outcomes. American journal of preventive medicine, 50(2), 129–135. https://doi.org/10.1016/j.amepre.2015.08.024

© 2024 Morency MM et al. *JAMA Network Open.*

© 2024 Morency MM et al. *JAMA Network Open.*
