# Supplementary material for: Structural Equality and Support Index in Early Childhood Education
Source: JAMA Netw Open. 2024 Aug 30;7(8):e2432050. doi: 10.1001/jamanetworkopen.2024.32050 (PMC11364995; doi:10.1001/jamanetworkopen.2024.32050)
Supplement: Supplement 2. — Data Sharing Statement [file jamanetwopen-e2432050-s002.pdf]

Data Sharing Statement

Morency. Structural Equality and Support Index in Early Childhood Education. *JAMA Netw Open*. Published August 30, 2024. doi:10.1001/jamanetworkopen.2024.32050

Data
Data available: Yes
Data types: Deidentified participant data, Data dictionary
How to access data: hcrc.umn.edu
When available: With publication

Supporting Documents
Document types: None

Additional Information
Who can access the data: Upon approved data sharing agreement.
Types of analyses: specified in data sharing agreement.
Mechanisms of data availability: proposal approval.
Any additional restrictions: None.
